# Distinct Molecular Signature of Earlobe Keloids: Integrated Transcriptomic Analysis of Extracellualr Matrix, Metalloproteinase, and Metabolic Pathways

**DOI:** 10.1007/s00266-026-06136-z

**Published:** 2026-07-07

**Authors:** Danielle Tafner, Andressa Marangoni Naccarato, Felipe Contoli Isoldi, Gabriel de Almeida Arruda Felix, Beatriz Ribeiro Nogueira, Rafael Leite Tavares de Morais, João Bosco Pesquero, Alfredo Gragnani

**Affiliations:** 1 Graduate Program in Translational Surgery, Universidade Federal de São Paulo (Unifesp), Rua Botucatu, 740 – Vila Clementino, São Paulo, SP 04023-900 Brazil; 2 Department of Biophysics, Universidade Federal de São Paulo, Rua Pedro de Toledo, 669, São Paulo, SP Brazil

**Keywords:** Keloid, Earlobe, Gene expression, Collagen, Metalloproteinases, Metabolism

## Abstract

**Background:**

Keloids are fibroproliferative scars that extend beyond the original wound margins and may cause pain, pruritus, and substantial psychosocial distress. Earlobe keloids are common, clinically distinctive lesions with high recurrence rates; however, the molecular mechanisms underlying their development remain incompletely characterized. Existing transcriptomic studies have largely focused on extracellular matrix genes, with limited integration of metabolism-related pathways.

**Aims:**

To characterize the integrated transcriptomic profile of earlobe keloids compared with site-matched normotrophic scars, allowing the characterization of a site-specific molecular profile, focusing on collagen and extracellular matrix, metalloproteinases, and metabolism-related genes, and to validate selected targets at the mRNA and protein levels.

**Methods:**

In this primary analytical case–control study, tissue samples were obtained from 11 earlobe keloids and 10 normotrophic earlobe scars in adults aged 18–50 years. After histopathological confirmation, total RNA was extracted and analyzed using next-generation RNA sequencing on the Ion S5 platform. Differentially expressed genes were identified using an absolute fold change ≥ 2 and a false discovery rate (FDR)-adjusted *p* value < 0.05, as calculated in Ion Reporter. Panels of collagen and extracellular matrix genes, metalloproteinase-related genes, and metabolism-related genes were selected for validation by quantitative real-time polymerase chain reaction, with ACTB and GAPDH as housekeeping genes. Relative expression was calculated using the 2^−ΔΔCt^ method. ADAM12 protein levels were quantified in tissue lysates using enzyme-linked immunosorbent assay. Statistical significance was set at *p* < 0.05.

**Results:**

RNA sequencing identified 20,809 genes across all samples, of which 664 were differentially expressed between keloid and normotrophic scar tissue. Earlobe keloids showed marked upregulation of collagen and extracellular matrix genes, including FN1, COL1A1, COL1A2, COL3A1, COL5A1, COL5A2, SPARC, LOX, and LOXL2, indicating reinforced matrix deposition and crosslinking. Metalloproteinase genes, such as ADAM12, MMP2, MMP11, MMP13, ADAMTS2, and ADAMTS12, together with TIMP1, were also overexpressed. Quantitative real-time PCR showed high concordance with transcriptomic fold changes, and ADAM12 protein was significantly increased in keloid tissue compared with normotrophic scars (*p* < 0.001). Metabolism-related genes, CPXM1, GALNT5, CHST6, and NOX4, were overexpressed, whereas ACADM, LEPR, APOC1, THRSP, and AWAT2 were downregulated, suggesting altered glycosylation, oxidative stress, and disturbed lipid metabolism in earlobe keloids.

**Conclusion:**

Earlobe keloids display a site-specific molecular profile characterized by coordinated dysregulation of collagen, metalloproteinases, and metabolism-related genes. The convergence of extracellular matrix accumulation, protease–antiprotease imbalance, and metabolic reprogramming supports the concept of keloids as metabolically active fibrotic tumors and identifies targets, such as ADAM12 and NOX4, for future translational studies. These findings reinforce the concept of anatomical site-dependent keloid biology and support targeted therapeutic approaches.

**Level of Evidence III:**

This journal requires that authors assign a level of evidence to each article. For a full description of these Evidence-Based Medicine ratings, please refer to the Table of Contents or the online Instructions to Authors www.springer.com/00266.

## Introduction

Keloids are benign fibroproliferative scars that extend beyond the boundaries of the original wound and fail to regress spontaneously [1, 2]. They may cause pain, pruritus, and considerable psychological distress, particularly when located in exposed areas [3, 4]. The earlobe is a frequent site of keloid formation after piercing, and lesions at this location are notable for their cosmetic and psychosocial impact and high recurrence rates despite treatment [5–7].

Given the emerging evidence that keloid biology varies across anatomical sites, focusing on a single, clinically relevant location, such as the earlobe, may reduce biological heterogeneity and enable more precise characterization of molecular mechanisms [5–7].

Keloid pathogenesis involves sustained extracellular matrix deposition, chronic inflammation, and aberrant fibroblast proliferation. Transcriptomic and histological studies have highlighted the dysregulation of genes related to collagen synthesis, matrix organization, and metalloproteinase activity. Collagens, such as COL1A1 and COL3A1, fibronectin (FN1), and matricellular proteins, including SPARC, are consistently overexpressed in keloid tissue compared to that in normal skin or normotrophic scars [8, 9]. Transforming growth factor-beta signaling and tissue inhibitors of metalloproteinases contribute to the imbalance between matrix production and degradation [8, 10–12].

Anatomical site appears to influence keloid biology. The distribution of keloids and hypertrophic scars varies across the body, with the earlobe, presternal region, and shoulders being among the most commonly affected sites [5, 6]. The earlobe differs from other keloid-prone areas in terms of tension, biomechanics, and adnexal density, and there is evidence that fibroblasts retain a site-specific transcriptomic identity, including distinct patterns of collagen gene expression in three-dimensional culture and differential collagen production at the growing margin of keloids [13, 14]. These observations support the need to study earlobe keloids in comparison with site-matched normotrophic scars rather than control tissue from unrelated anatomical locations [7].

In addition to ECM remodeling, emerging data suggest that metabolic reprogramming also contributes to fibrotic skin diseases. Lipid metabolism appears to be altered in keloids, with changes in lipid composition and signaling pathways implicated in scar formation [15]. Keloid fibroblasts exhibit evidence of increased oxidative stress and altered glucose metabolism under hypoxic conditions and transforming growth factor-beta stimulation, including activation of EGR1 and NOX4 signaling [16, 17]. Adipokines, such as leptin, have been associated with exuberant scar formation, and genes involved in glycosaminoglycan metabolism, such as CHST6, may contribute to abnormal dermal matrix composition [18, 19]. Meanwhile, multi-omic and multi-ancestry studies have identified genetic susceptibility loci and regulatory networks relevant to fibrosis and inflammation in keloid tissue [10, 11, 20–23]. Despite these advances, few studies have provided an integrated view linking collagen, metalloproteinases, and metabolic pathways in site-specific earlobe keloids using a single patient cohort and a unified analytic pipeline. The present study aimed to perform an integrated transcriptomic and quantitative validation analysis of earlobe keloids compared with normotrophic earlobe scars. We focused on three interconnected gene groups: collagen and ECM genes, metalloproteinases and related regulators, and metabolism-related genes. By analyzing these pathways within the same cohort, we sought to delineate a site-specific molecular signature that may inform future mechanistic and therapeutic studies.

## Methods

### Study Design and Participants

This was a primary analytical case–control study including adult patients aged 18–50 years who underwent surgical excision of earlobe keloids at a tertiary academic center. Normotrophic earlobe scars from patients undergoing elective procedures were used as site-matched controls. All participants provided written informed consent, and the study was approved by the Institutional Ethics Committee. The study was conducted in accordance with the STROBE guidelines for observational studies.

The inclusion criteria for the keloid group were a clinically diagnosed earlobe keloid of at least 12 months duration, absence of specific keloid treatment in the preceding 6 months, and willingness to undergo surgical excision. The control group included normotrophic scars on the earlobe resulting from earrings, piercings, or stretchers. The exclusion criteria for both groups were collagen diseases, previous or current malignancy, systemic corticosteroid use or chemotherapy, menopause, pregnancy, breastfeeding, active cutaneous infection, and decompensated systemic comorbidities.

### Ethics

The study protocol was approved by the Institutional Research Ethics Committee (details withheld for anonymous review). All procedures complied with the principles of the Declaration of Helsinki. Written informed consent was obtained from all participants for participation and tissue collection.

### Histopathological Confirmation and Tissue Sampling

All tissue samples underwent histopathological confirmation before molecular analysis. Keloid specimens were confirmed based on compatible clinical history and histological features, including dense hyalinized collagen bundles, fibroproliferative dermal expansion, and scar architecture extending beyond the expected pattern of normotrophic repair. Normotrophic scar samples were confirmed by the absence of keloid-like dermal expansion and prominent hyalinized collagen bundles.

Tissue sampling was performed using excised earlobe lesions and site-matched normotrophic earlobe scars. For keloids, representative tissue was obtained from the excised lesion while avoiding grossly necrotic, infected, or cauterized areas. The samples included dermal scar tissue and the adjacent overlying soft tissue, as present in the surgical specimen. Because bulk RNA sequencing was performed on whole tissue samples, potential differences in tissue composition, including fibroblast density, inflammatory infiltrate, vascularity, epidermal contribution, adnexal structures, and extracellular matrix content, were considered when interpreting the transcriptomic findings.

### RNA Extraction and Transcriptome Sequencing

Approximately 30 mg of each frozen tissue sample was disrupted using a TissueLyser system (Qiagen, Hilden, Germany). Total RNA was extracted using the RNeasy Fibrous Tissue Mini Kit (Qiagen, Hilden, Germany) according to the manufacturer’s instructions. RNA quality and sequencing metrics were assessed before downstream analysis. Library preparation and sequencing quality were evaluated according to the Ion AmpliSeq Transcriptome workflow. Quality control parameters included RNA concentration, library quantification, sequencing output, assay-specific quality metrics, and sample-level performance in the Ion Reporter. RNA integrity was assessed prior to library preparation according to the manufacturer’s workflow recommendations. Samples that did not meet the platform quality requirements were excluded from downstream analysis. Low-quality or low-expression targets were filtered according to the Ion Reporter transcriptome analysis workflow before differential expression testing.

Complementary DNA synthesis and library preparation were performed using the Ion AmpliSeq Transcriptome Human Gene Expression Kit (Invitrogen, Carlsbad, CA, USA). Libraries were quantified using the Ion Library TaqMan Quantitation Kit and loaded onto the Ion Chef system. Sequencing was performed using an Ion S5 platform (Invitrogen, Carlsbad, CA, USA). Data processing and differential expression analysis were performed using the Ion Torrent Suite and Ion Reporter software. Differential expression analysis was conducted using the Ion Reporter’s built-in statistical workflow. Multiple testing correction was performed using false discovery rate (FDR)-adjusted *p* values. Genes were considered differentially expressed only when they met both predefined criteria: absolute fold change ≥ 2 and FDR-adjusted p-value < 0.05.

All samples were processed using the same library preparation protocol and sequencing platform. No additional covariates were included in the differential expression model because of the modest sample size and absence of statistically significant differences in baseline demographic variables between the groups.

### Gene Selection for Validation

From the list of differentially expressed genes, we selected three panels for validation based on biological relevance and prior literature.

Collagen and extracellular matrix genes: FN1, COL1A1, COL1A2, COL3A1, COL5A1, COL5A2, SPARC, BGN, COL6A1, COL8A1, COL10A1, LOX, and LOXL2, which have been implicated in matrix organization and stiffness in keloid and fibrotic tissues [8, 9, 24]

Metalloproteinases and related regulators: ADAM12, MMP2, MMP11, MMP13, MMP23B, ADAMTS2, ADAMTS12, and TIMP1, given the recognized role of matrix metalloproteinases, ADAM family members, and tissue inhibitors of metalloproteinases in scar remodeling and fibrosis [8, 10–12, 25–27].

Metabolism-related genes: CPXM1, GALNT5, CHST6, NOX4, ACADM, LEPR, APOC1, THRSP, and AWAT2. CPXM1 is a secreted collagen-binding glycoprotein [28], GALNT5 and CHST6 are involved in O-glycosylation and glycosaminoglycan sulfation [19], and NOX4 mediates reactive oxygen species generation downstream of EGR1.

### Quantitative Real-Time PCR

Quantitative real-time PCR was performed using TaqMan assays (Applied Biosystems, Foster City, CA, USA) in accordance with the manufacturer’s protocol. Complementary DNA was synthesized from extracted RNA using a high-capacity cDNA synthesis kit (Applied Biosystems). ACTB and GAPDH were used as housekeeping genes. Relative expression was calculated using the 2^−ΔΔCt^ method, and results are presented as fold change with 95 percent confidence intervals [29]

### ADAM12 Protein Quantification

ADAM12 protein levels were measured in tissue lysates using a human ADAM12 ELISA kit (Abcam, Cambridge, UK). Total protein concentration was determined using the Bradford assay. Samples and standards were processed in duplicate according to the manufacturer’s instructions, and absorbance was measured at 450 nm using a microplate reader. ADAM12 concentrations were derived from standard curves and normalized to total protein content.

### Statistical Analysis

Baseline demographic and clinical variables are presented as means and standard deviations for continuous data and counts and percentages for categorical data. Normality was assessed using the Shapiro–Wilk test. Group comparisons were performed using the unpaired Student’s t-test or Mann–Whitney U-test for continuous variables and the Chi-squared test or Fisher’s exact test for categorical variables.

For RNA sequencing, differential expression analysis was performed using Ion Reporter, and multiple testing correction was applied using FDR-adjusted *p* values. Genes were considered differentially expressed when they met both predefined criteria: absolute fold change ≥ 2 and FDR-adjusted *p* value < 0.05.

For targeted quantitative real-time PCR validation, relative expression was calculated using the 2^−ΔΔCt^ method and expressed as fold change with 95% confidence intervals [29]. Because the RT-qPCR analyses involved predefined biologically selected validation targets rather than transcriptome-wide discovery testing, Bonferroni correction was applied only to simultaneous multiple comparisons performed within the same predefined biological validation panel. These analyses were interpreted as validation-level analyses of predefined gene panels selected from the RNA-seq results and prior biological relevance, rather than as independent transcriptome-wide discovery analyses.

ADAM12 protein levels measured by ELISA were compared between groups using the appropriate parametric or non-parametric test according to data distribution. Statistical significance was set at *p* < 0.05. Analyses were performed using IBM SPSS Statistics version 22 (IBM Corp., Armonk, NY, USA).

## Results

### Baseline Characteristics

Twenty-one participants were included: 11 in the earlobe keloid group and 10 in the normotrophic scar group. The mean age was similar between the groups, and there were no significant differences in sex distribution or self-reported ethnicity.

Table 1 summarizes the demographic and clinical characteristics of the two groups.

**Table 1:** Demographic and clinical characteristics of patients with earlobe keloids and normotrophic earlobe scars

Variable	Keloid group (KG, *n* = 11)	Control group (CG, *n* = 10)	Total (*N* = 21)	*p* value
Age, years (mean ± SD)	25.4 ± 7.7	28.1 ± 3.7	26.7 ± 6.3	0.21
Gender, Female, *n* (%)	5 (45.5%)	5 (50.0%)	10 (47.6%)	0.84
Gender, Male, *n* (%)	6 (54.5%)	5 (50.0%)	11 (52.4%)	
Ethnicity, White, *n* (%)	5 (45.5%)	7 (70.0%)	12 (57.1%)	0.32
Ethnicity, Black, n (%)	2 (18.2%)	0 (0.0%)	2 (9.5%)	
Ethnicity, Mixed, *n* (%)	4 (36.3%)	3 (30.0%)	7 (33.3%)	

*KG* keloid group, *CG* control group, *SD* standard deviation

### Global Transcriptomic Profile

RNA sequencing identified 20,809 genes expressed across all samples. Of these, 664 genes met the predefined criteria for differential expressions, defined as an absolute fold change ≥ 2 and an FDR-adjusted *p* value < 0.05. Among these, 345 genes were upregulated, and 319 were downregulated in earlobe keloids compared to normotrophic scars. Fold-change values ranged from approximately + 59.9 to − 10.87, indicating marked transcriptional reprogramming in earlobe keloids.

**Fig. 1:**
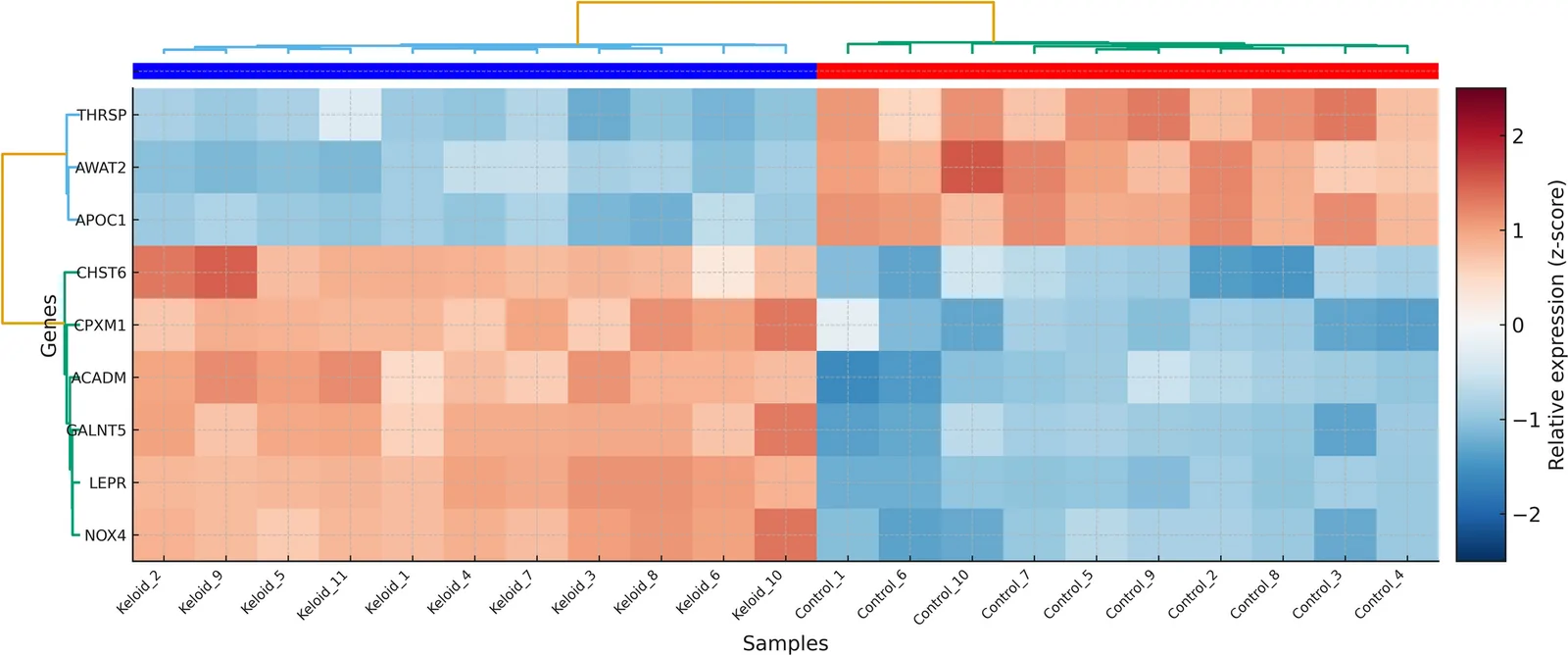
Hierarchical clustering heatmap of selected differentially expressed genes related to collagen and extracellular matrix, metalloproteinases, and metabolism in earlobe keloids compared with normotrophic earlobe scars. Each column represents an individual sample, and each row represents a gene. Red indicates relative upregulation, and blue indicates relative downregulation in keloid tissue. *KG* keloid group, *CG* control group

A hierarchical clustering heatmap of the selected genes revealed clear segregation between keloid and normotrophic scar samples based on the expression of collagen, metalloproteinase, and metabolism-related genes (Fig. 1).

### Collagen and Extracellular Matrix Genes

Earlobe keloids showed marked overexpression of several collagen and extracellular matrix genes. FN1 exhibited the highest fold change among the selected targets, followed by COL1A1, COL1A2, COL3A1, COL5A1, and COL5A2. SPARC, BGN, COL6A1, COL8A1, and COL10A1 were also significantly upregulated. LOX and LOXL2, enzymes involved in collagen crosslinking, were consistently overexpressed in patients.

**Fig. 2:**
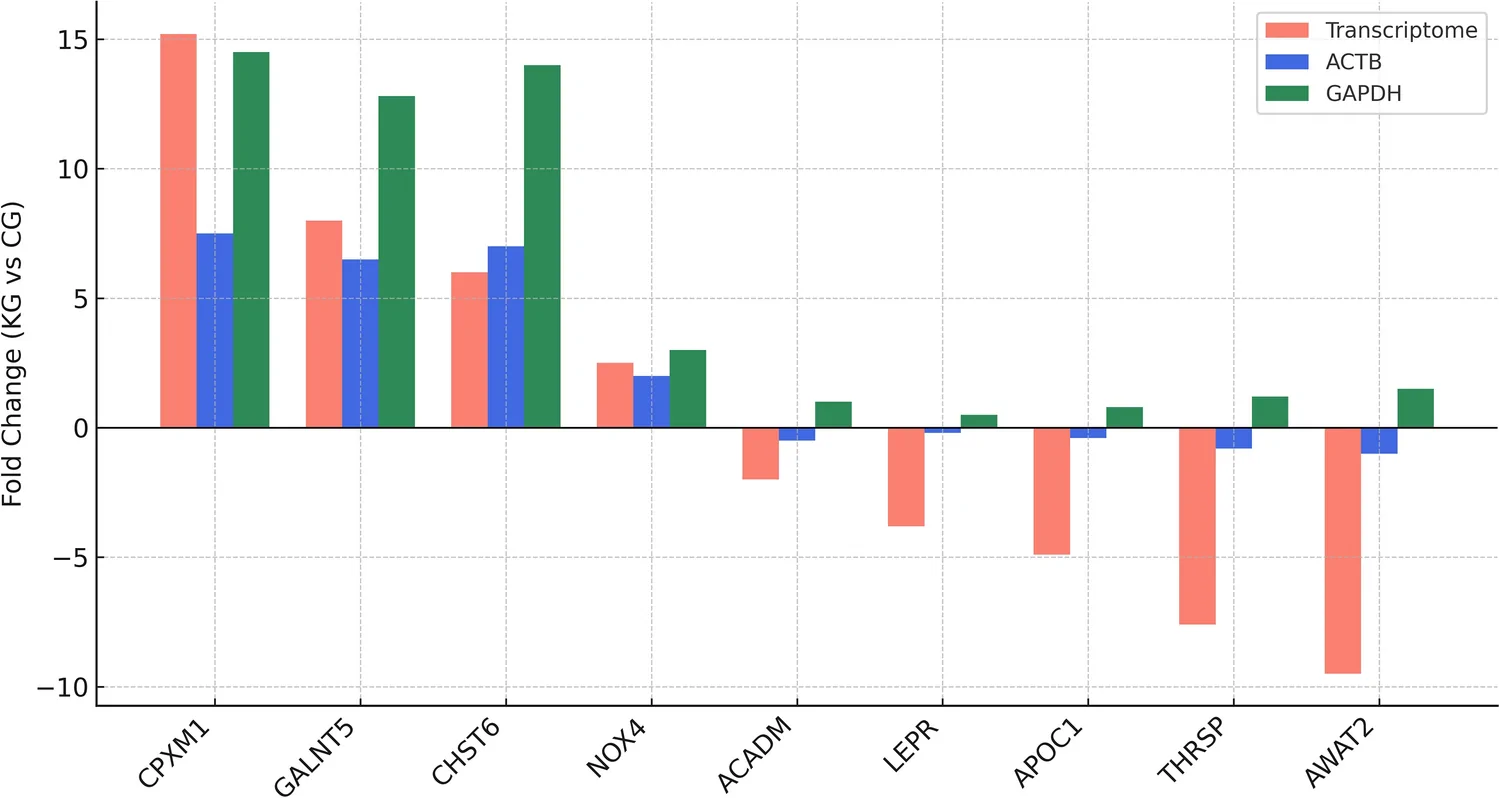
Collagen and extracellular matrix-related gene expression in earlobe keloids compared with normotrophic earlobe scars by RNA sequencing and quantitative real-time PCR. Bars represent fold change (KG vs. CG) for FN1, COL1A1, COL1A2, COL3A1, COL5A1, COL5A2, SPARC, BGN, COL6A1, COL8A1, COL10A1, LOX, and LOXL2. *FC* fold change, *RT-PCR* real-time polymerase chain reaction, *ECM* extracellular matrix

Quantitative real-time PCR (RT-qPCR) validation confirmed the direction and magnitude of the fold changes obtained by RNA sequencing for all collagen-related genes. Table 2 shows the transcriptomic and quantitative real-time PCR fold-change values for collagen and extracellular matrix targets, and Fig. 2 illustrates the concordant expression profiles.

**Table 2:** Fold-change values for collagen and extracellular matrix-related genes in earlobe keloids compared with normotrophic earlobe scars by RNA sequencing and quantitative RT-PCR

Gene	Transcriptome FC	RT-PCR FC (ACTB)	RT-PCR FC (GAPDH)
FN1	59.9	15.33	31.76
SPARC	14.79	8.92	18.48
BGN	4.96	3.04	6.31
COL10A1	10.15	5.3	10.98
COL5A2	20.94	15.44	31.99
COL5A1	22.37	10.84	22.46
COL3A1	27.27	13.95	28.89
COL1A2	27.96	30.87	63.96
COL1A1	47.78	26.93	55.79
COL8A1	8	13.62	28.22
COL6A1	5.9	4.64	9.62
LOX	6.56	4.47	9.26
LOXL2	9.25	9.65	19.99

*FC* fold change, *RT-PCR* real-time polymerase chain reaction; ACTB and GAPDH were used as housekeeping genes, and relative expression was calculated using the 2^−ΔΔCt^ method

### Metalloproteinases and ADAM12 Protein Expression

Several metalloproteinase genes were significantly dysregulated in earlobe keloids. ADAM12 showed the most striking results, with high fold-change values in both transcriptomic and quantitative real-time PCR analyses. MMP2, MMP11, MMP13, MMP23B, ADAMTS2, and ADAMTS12 were also upregulated, together with TIMP1 [8, 10–12, 25–27].

**Fig. 3:**
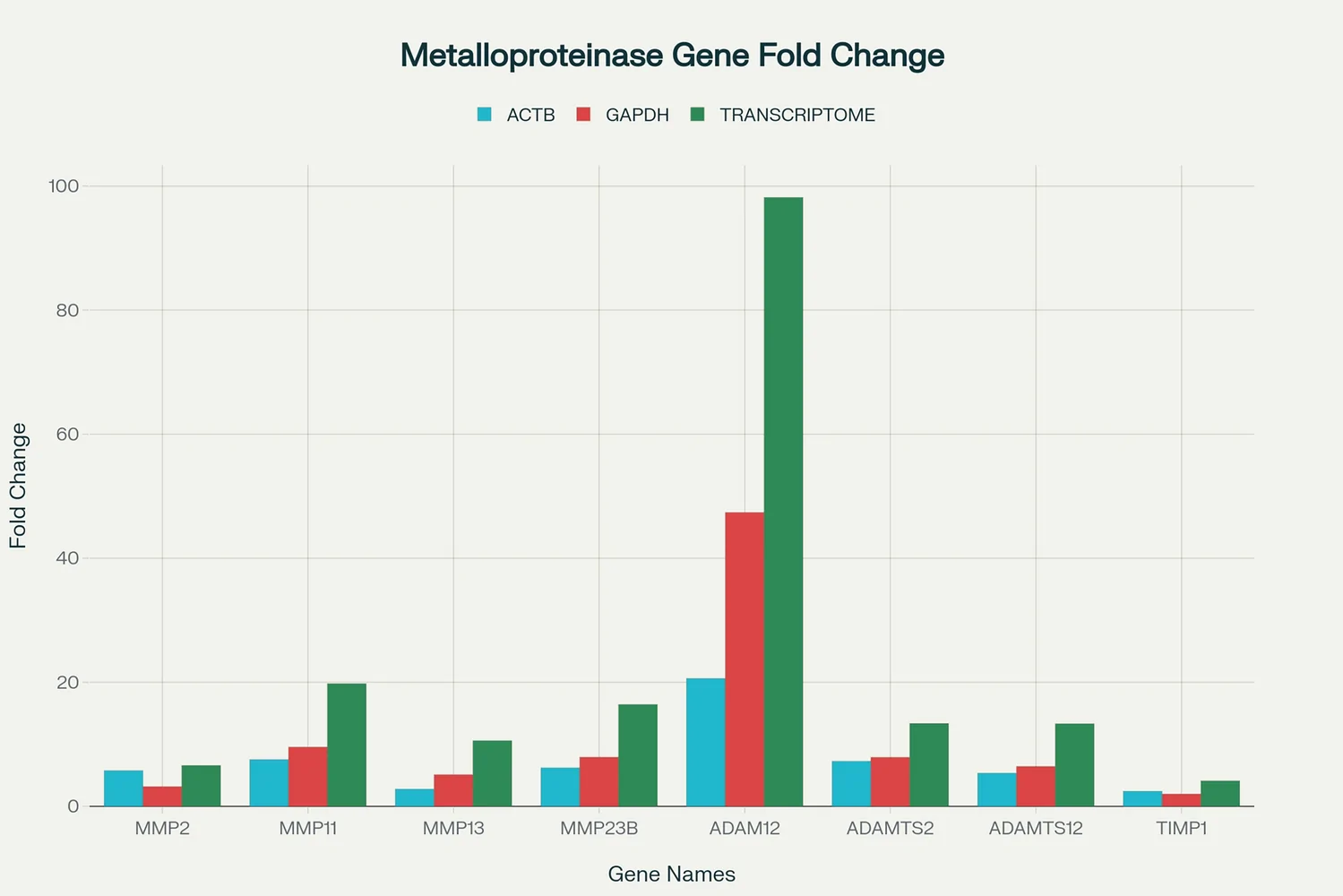
Metalloproteinase-related gene expression in earlobe keloids compared with normotrophic earlobe scars by RNA sequencing and quantitative real-time PCR. Bars represent fold changes (KG vs. CG) for MMP2, MMP11, MMP13, MMP23B, ADAM12, ADAMTS2, ADAMTS12, and TIMP1.*FC* fold change, *RT-PCR* real-time polymerase chain reaction

Quantitative real-time PCR validation confirmed these patterns (Table 3), showing robust overexpression of ADAM12 and associated proteases in keloid tissue compared with normotrophic scars. Figure 3 summarizes the expression data for metalloproteinase-related genes.

**Table 3:** Fold-change values for metalloproteinase-related genes in earlobe keloids compared with normotrophic earlobe scars by RNA sequencing and quantitative RT-PCR

Gene	Transcriptome FC	RT-PCR FC (ACTB)	RT-PCR FC (GAPDH)
MMP2	5.75	3.18	6.60
MMP11	7.55	9.55	19.79
MMP13	2.77	5.11	10.59
MMP23B	6.22	7.92	16.41
ADAM12	20.61	47.38	98.17
ADAMTS2	7.27	7.91	13.38
ADAMTS12	5.36	6.43	13.32
TIMP1	2.45	1.99	4.12

*FC* fold change, *RT-PCR* real-time polymerase chain reaction; ACTB and GAPDH were used as housekeeping genes, and relative expression was calculated using the 2^−ΔΔCt^ method.

**Fig. 4:**
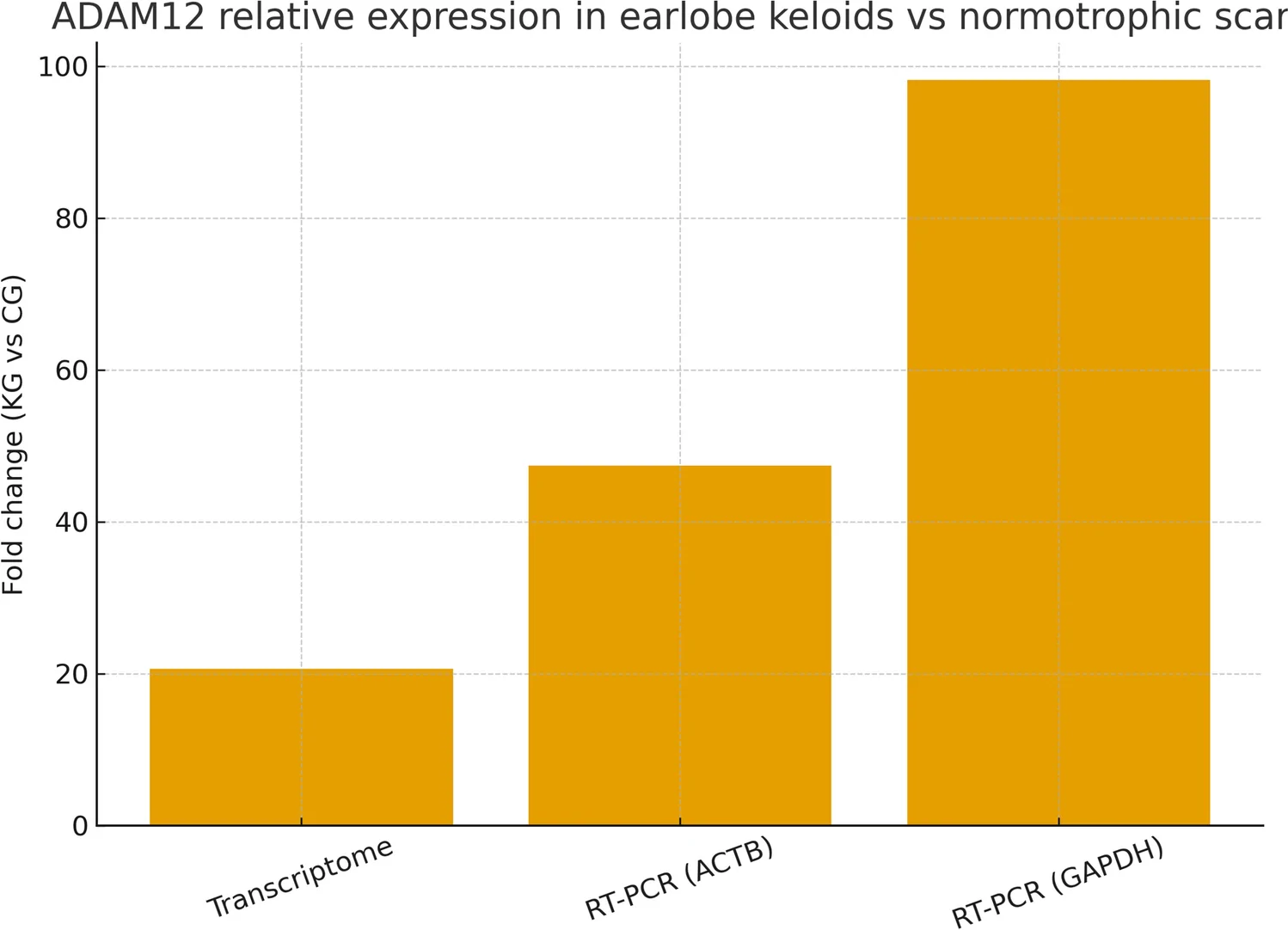
ADAM12 mRNA expression in earlobe keloids compared with normotrophic earlobe scars. Bars represent fold change (KG vs. CG) obtained from transcriptome analysis and quantitative real-time PCR normalized to ACTB and GAPDH using the 2^−ΔΔCt^ method. *FC* fold change, *RT-PCR* real-time polymerase chain reaction

**Fig. 5:**
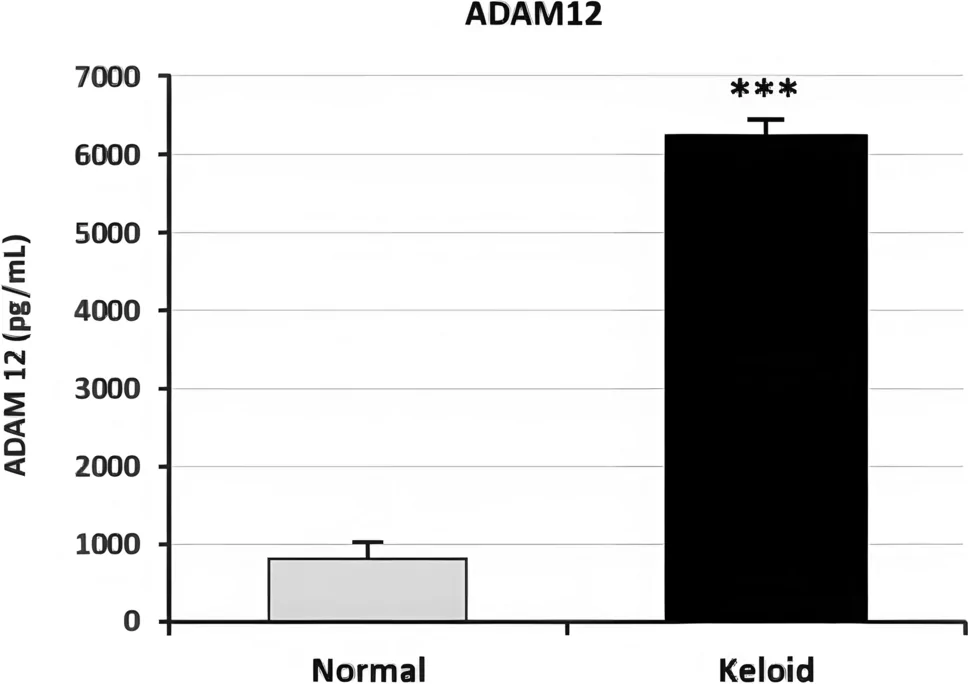
ADAM12 protein concentration in tissue lysates from earlobe keloids and normotrophic earlobe scars, measured by enzyme-linked immunosorbent assay. Bars represent ADAM12 levels normalized to total protein content. Differences between the groups were statistically significant (*p* < 0.001). *ELISA* enzyme-linked immunosorbent assay, *KG* keloid group, *CG* control group

ADAM12 protein levels, measured by ELISA, were significantly higher in keloid tissue than in normotrophic scars (*p* < 0.001) (Fig. 4), indicating that transcriptional upregulation is accompanied by an increase in protein expression. Figure 5 shows the distribution of ADAM12 protein levels between the two groups.

### Metabolism-Related Genes

Metabolism-related genes displayed a distinct pattern of dysregulation. CPXM1, GALNT5, CHST6, and NOX4 were overexpressed in earlobe keloids compared to normotrophic scars. These genes are implicated in collagen binding and glycosylation patterns that could influence matrix stability and cell matrix interactions, as well as reactive oxygen species generation, suggesting enhanced matrix remodeling, abnormal glycosylation, and oxidative stress in keloid tissue [15, 17, 18, 22, 23].

In contrast, ACADM, LEPR, APOC1, THRSP, and AWAT2 were consistently downregulated. These targets are involved in fatty acid oxidation, leptin signaling, lipid transport, lipid biosynthesis, and epidermal barrier function, respectively, indicating impaired lipid metabolism and altered immune–endocrine regulation [15–18, 22, 23].

Quantitative real-time PCR validation demonstrated a strong concordance with transcriptomic data for all metabolism-related genes. Table 4 shows the quantitative real-time PCR results for the metabolism-related genes.

**Table 4:** Evidence matrix of included publications according to study model, mechanistic domain, extracellular matrix outcome, and contribution to the scoping review

Gene	Function	RT-PCR FC (95% CI)	*p* value
CPXM1	Collagen-binding glycoprotein	11.2 (8.9–13.5)	< 0.01
GALNT5	O-glycosylation enzyme	9.8 (7.5–12.1)	< 0.01
CHST6	GAG sulfation	10.9 (8.1–13.7)	< 0.01
NOX4	Oxidative stress	2.8 (1.9–3.7)	0.02
ACADM	Fatty acid oxidation	− 2.0 (− 3.0 to − 1.1)	0.03
LEPR	Leptin signaling	− 3.8 (− 5.4 to − 2.2)	0.01
APOC1	Lipid transport	− 4.9 (− 6.5 to − 3.2)	0.02
THRSP	Lipid biosynthesis	− 7.6 (− 9.8 to − 5.4)	< 0.01
AWAT2	Barrier lipid synthesis	− 9.5 (− 12.3 to − 6.7)	< 0.01

*FC* fold change, *RT-PCR* real-time polymerase chain reaction, *CI* confidence interval, *ECM* extracellular matrix, *ROS* reactive oxygen species

## Discussion

This study demonstrates that earlobe keloids exhibit a distinct transcriptomic profile characterized by coordinated dysregulation of extracellular matrix, metalloproteinase, and metabolism-related pathways.

The use of site-matched controls strengthens internal validity by reducing confounding related to anatomical variability.

Notably, these findings should be interpreted in the context of bulk tissue transcriptomic profiling. Because RNA sequencing was performed on whole tissue rather than isolated cell populations or single-cell preparations, the observed gene expression patterns may reflect both true transcriptional changes within resident cells and differences in tissue composition between keloid and normotrophic scars. Variations in fibroblast density, inflammatory infiltrate, endothelial cells, epidermal contribution, adnexal structures, adipose tissue, and extracellular matrix abundance may contribute to the detected transcriptomic signal. Therefore, the present findings should be viewed as a tissue-level molecular signature of earlobe keloids rather than definitive evidence of cell-type-specific mechanisms.

The upregulation of collagen and extracellular matrix genes observed in this study is consistent with the previous work demonstrating increased collagen production and altered matrix organization in keloid fibroblasts [8, 9, 13, 24]. The concomitant overexpression of LOX and LOXL2 suggests enhanced collagen crosslinking, contributing to increased tissue stiffness and persistence of fibrotic lesions.

Metalloproteinase dysregulation further supports an imbalance between matrix synthesis and degradation. Increased expression of ADAM12, MMPs, and TIMP1 may reflect a microenvironment that favors persistent fibrosis, in line with emerging data on ADAM family members and matrix remodeling in keloid lesions [8, 10–12, 25–27]. Among these targets, ADAM12 showed particularly marked upregulation at both the transcript and protein levels, identifying it as a prominent component of the molecular signature observed in earlobe keloids. However, the present study could not determine whether ADAM12 acts as a driver of fibrosis, a marker of fibroproliferative activity, or a downstream consequence of the keloid microenvironment. Functional studies are required to determine whether ADAM12 directly contributes to fibroblast activation, extracellular matrix accumulation, recurrence risk, or treatment response.

In addition to structural remodeling, our findings highlight the role of metabolic reprogramming in keloid biology. The upregulation of NOX4 and related pathways is consistent with previous demonstrations of oxidative stress-mediated fibrosis [16, 17]. Alterations in lipid metabolism genes and adipokine signaling further support a model in which keloids represent a metabolically active fibrotic state [15, 17, 18, 22, 23].

The interpretation of metabolism-related genes requires particular caution. Changes in lipid metabolism, adipokine signaling, oxidative stress, and glycosylation-related genes may reflect altered fibroblast biology; however, they may also be influenced by differences in tissue composition, including adipose tissue, inflammatory cells, vascularity, and epidermal or adnexal components. In addition, because this was a modest single-center cohort focused exclusively on earlobe lesions, these findings should not be generalized to all keloid phenotypes without validation in larger cohorts and keloids from other anatomical sites.

These findings align with recent multi-omic and multi-ancestry studies that have identified regulatory networks associated with fibrosis and inflammation in keloid tissue [10, 11, 20–23].

From a translational perspective, the convergence of extracellular matrix, metalloproteinase, and metabolism-related abnormalities suggests several pathways that may warrant future mechanistic studies. However, the present findings should be considered hypothesis-generating. Although ADAM12, NOX4, and collagen crosslinking pathways may represent biologically plausible targets, this study did not test whether modulation of these pathways alters fibroblast behavior, scar progression, recurrence risk, or treatment response. Future studies integrating transcriptomics with proteomics, metabolomics, spatial transcriptomics, and functional assays are required before these targets can be considered clinically actionable.

## Limitations

This study has several limitations. First, the sample size was modest and derived from a single center, limiting the statistical power and external validity. Although the use of site-matched earlobe controls improves internal validity by reducing anatomical heterogeneity, the findings cannot be directly extrapolated to keloids from other anatomical sites, such as the chest, shoulders, or presternal region, where mechanical tension and local skin characteristics differ [5, 6].

Second, hypertrophic scars and keloids from other anatomical sites were not included in the comparison groups. This choice was deliberate to minimize biological heterogeneity in an initial site-specific transcriptomic analysis; however, it prevents the direct determination of whether the identified molecular signature is specific to earlobe keloids, to keloids more broadly, or to pathological scarring in general. Future studies should incorporate multi-arm designs that include hypertrophic scars, normotrophic scars, and keloids from different anatomical regions [9].

Third, RNA sequencing was performed on bulk tissue samples rather than isolated fibroblasts, sorted cell populations, single-cell preparations, or spatially resolved tissue sections. Therefore, some gene expression differences may reflect variations in tissue composition, including fibroblast density, inflammatory infiltration, vascularity, epidermal contribution, adnexal structures, adipose tissue, and extracellular matrix abundance.

Fourth, although the selected targets were validated by quantitative real-time PCR, and ADAM12 was validated at the protein level by ELISA, there was no independent validation cohort. The study did not include functional assays to determine whether the observed molecular alterations directly affect fibroblast behavior, extracellular matrix production, scar progression, recurrence, or treatment response.

Finally, although baseline demographic variables did not differ significantly between the groups, the modest sample size limited adjustment for potential confounders, such as sex, ethnicity, skin phototype, clinical history, and technical batch effects. Future studies integrating transcriptomic analysis with proteomic analysis, metabolomic analysis, spatial transcriptomic analysis, and functional models are needed to establish causal links and to determine whether ADAM12, NOX4, collagen crosslinking enzymes, or metabolism-related pathways can be therapeutically targeted.

## Conclusion

Earlobe keloids exhibit a distinct molecular profile characterized by coordinated dysregulation of collagen, metalloproteinase, and metabolism-related genes. These findings support the concept of site-specific scar biology and may contribute to the development of targeted therapeutic strategies.

## Data Availability

The datasets generated and analyzed during the current study are available from the corresponding author upon reasonable request. De-identified processed gene expression data can be provided for academic purposes, subject to institutional and ethical restrictions.

## References

[1] Marneros AG, Krieg T. Keloids: clinical diagnosis, pathogenesis, and treatment options. J Dtsch Dermatol Ges. 2004;2:905–13. 10.1046/j.1439-0353.2004.04077.x 16281608

[2] Slemp AE, Kirschner RE. Keloids and scars: a review of their pathogenesis, risk factors, and management. Curr Opin Pediatr. 2006;18:396–402. 10.1097/01.mop.0000236389.41462.ef 16914994

[3] Hunasgi S, Koneru A, Vanishree M, Shamala R. Keloid: a case report and review of pathophysiology and differences between keloid and hypertrophic scars. J Oral Maxillofac Pathol. 2013;17:116–20. 10.4103/0973-029X.110701 PMC3687166 23798844

[4] Andrews JP, Marttala J, Macarak E, Rosenbloom J, Uitto J. Keloids: the paradigm of skin fibrosis pathomechanisms and treatment. Matrix Biol. 2016;51:37–46. 10.1016/j.matbio.2016.01.013 PMC4842154 26844756

[5] Hochman B, Farkas CB, Isoldi FC, Ferreira LM. Keloid and hypertrophic scar distribution according to Fitzpatrick skin phototypes. Rev Bras Cir Plást. 2012;27:185–9.

[6] Hochman B, Isoldi FC, Furtado F, Ferreira LM. New approach to the understanding of keloid: psychoneuroimmune endocrine aspects. Clin Cosmet Investig Dermatol. 2015;8:67–73. 10.2147/CCID.S49195 PMC4329995 25709489

[7] Hochman B, Isoldi FC, Silveira TS, Borba GC, Ferreira LM. Does ear keloid formation depend on the type of earrings or piercing jewellery? Australas J Dermatol. 2015;56:e77–9. 10.1111/ajd.12190 25715880

[8] Ogawa R. Keloid and hypertrophic scars are the result of chronic inflammation in the reticular dermis. Int J Mol Sci. 2017;18:606. 10.3390/ijms18030606 PMC5372622 28287424

[9] Ojeh N, Bharatha A, Gaur U, Forde AL. Keloids: current and emerging therapies. Scars Burn Heal. 2020;6:2059513120940499. 10.1177/2059513120940499 PMC7418256 32844039

[10] Limandjaja GC, Niessen FB, Scheper RJ, Gibbs S. The keloid disorder: heterogeneity, histopathology, mechanisms and models. Front Cell Dev Biol. 2020;8:360. 10.3389/fcell.2020.00360 PMC7264387 32528951

[11] Cohen AJ, Nikbakht N, Uitto J. Keloid disorder: genetic basis, gene expression profiles, and immunological modulation of the fibrotic processes in the skin. Cold Spring Harb Perspect Biol. 2023. 10.1101/cshperspect.a041245. PMC10317059 36411063

[12] Fang X, Wang Y, Chen H, Yan Z, Jin S, Wu Y, et al. Hypertrophic scarring and keloids: epidemiology, molecular pathogenesis, and therapeutic interventions. MedComm. 2020;6:e70381. 10.1002/mco2.70381 PMC12495451 41049267

[13] Sato M, Ishikawa O, Miyachi Y. Distinct patterns of collagen gene expression are seen in normal and keloid fibroblasts grown in three-dimensional culture. Br J Dermatol. 1998;138:938–43. 10.1046/j.1365-2133.1998.02258.x 9747353

[14] Syed F, Ahmadi E, Iqbal SA, Singh S, McGrouther DA, Bayat A. Fibroblasts from the growing margin of keloid scars produce higher levels of collagen I and III compared with intralesional and extralesional sites: clinical implications for lesional site-directed therapy. J Invest Dermatol. 2011;131:722–30. 10.1111/j.1365-2133.2010.10048.x 20849516

[15] Huang C, Ogawa R. Roles of lipid metabolism in keloid development. Lipids Health Dis. 2013;12:60. 10.1186/1476-511X-12-60 PMC3653711 23634948

[16] Qin H, Zhang L, Li M, Liu Y, Sun S, Nie W, et al. EGR1 and NOX4 pathway regulates oxidative stress and further facilitates fibrosis progression in keloids responses to TGF-beta 1. J Dermatol Sci. 2022;108:138–45. 10.1016/j.jdermsci.2022.12.009 36608994

[17] Wang Q, Wang P, Qin Z, Yang X, Pan B, Nie F, et al. Altered glucose metabolism and cell function in keloid fibroblasts under hypoxia. Redox Biol. 2021;38:101815. 10.1016/j.redox.2020.101815 PMC7718484 33278780

[18] Seleit I, Bakry OA, Samaka RM, Tawfik AS. Immunohistochemical evaluation of leptin expression in wound healing: a clue to exuberant scar formation. Appl Immunohistochem Mol Morphol. 2016;24:285–91. 10.1097/PAI.0000000000000187 26258753

[19] Singh S, Das S, Kannabiran C, Jakati S, Chaurasia S. Macular corneal dystrophy: an updated review. Curr Eye Res. 2021;46:765–70. 10.1080/02713683.2020.1849727 33171054

[20] Greene CA, Hampton G, Jaworski J, Shuey MM, Khan A, Luo Y, et al. Multi-ancestry meta-analysis of keloids uncovers novel susceptibility loci in diverse populations. Nat Commun. 2025;16:7770. 10.1038/s41467-025-62945-x PMC12368108 40835838

[21] Huang J, Zhou X, Wang W, Zhou G, Zhang WJ, Gao Z, et al. Combined analyses of RNA sequence and Hi-C along with GWAS loci: a novel approach to dissect keloid disorder genetic mechanism. PLoS Genet. 2022;18:e1010168. 10.1371/journal.pgen.1010168 PMC9202908 35709140

[22] Li D, Li M, Xue J, Zhou H, Tang J, et al. Integrated bioinformatics analysis reveals novel key biomarkers and potential candidate small molecule drugs in keloids. Int J Mol Med. 2020;45:1305–18.

[23] Shim J, Oh SJ, Yeo E, Park JH, Bae JH, Kim SH, et al. Integrated analysis of single-cell and spatial transcriptomics in keloids: highlights on fibrovascular interactions in keloid pathogenesis. J Invest Dermatol. 2022;142:2128–39. 10.1016/j.jid.2022.01.017 35123990

[24] Marttala J, Macarak E, Rosenbloom J, Uitto J. Keloids: developmental and genetic aspects. Matrix Biol. 2016;51:37–46. 10.1016/j.matbio.2016.01.013 PMC4842154 26844756

[25] Xiao Y, Lin N, Chen H, Xiao H. MeCP2 promotes keloid progression by regulating ADAM12 expression and Wnt beta-catenin pathway. Arch Dermatol Res. 2025;317:621. 10.1007/s00403-025-04131-z 40119949

[26] Rath M, Pitiot A, Kirr M, Fröhlich W, Plosnita B, Schliep S, et al. Multi-antigen imaging reveals inflammatory dendritic cells, ADAM17 and neprilysin as effectors in keloid formation. Int J Mol Sci. 2021;22:9326. 10.3390/ijms22179417 PMC8430546 34502327

[27] Kim H, Anggradita LD, Lee SJ, Hur SS, Bae J, Hwang NS, et al. Ameliorating fibrotic phenotypes of keloid dermal fibroblasts through an epidermal growth factor-mediated extracellular matrix remodelling. Int J Mol Sci. 2021;22:1657. 10.3390/ijms22042198 PMC7926382 33672186

[28] Kim YH, O’Neill HM, Whitehead JP. Carboxypeptidase X 1 (CPX1) is a secreted collagen-binding glycoprotein. Biochem Biophys Res Commun. 2015;468:894–9. 10.1016/j.bbrc.2015.11.053 26603934

[29] Livak KJ, Schmittgen TD. Analysis of relative gene expression data using real-time quantitative PCR and the 2^−ΔΔCt^ method. Methods. 2001;25:402–8. 10.1006/meth.2001.1262 11846609

